# Acquisition of IgG to ICAM-1-Binding DBLβ Domains in the Plasmodium falciparum Erythrocyte Membrane Protein 1 Antigen Family Varies between Groups A, B, and C

**DOI:** 10.1128/IAI.00224-19

**Published:** 2019-09-19

**Authors:** Rebecca W. Olsen, Gertrude Ecklu-Mensah, Anja Bengtsson, Michael F. Ofori, Kwadwo A. Kusi, Kwadwo A. Koram, Lars Hviid, Yvonne Adams, Anja T. R. Jensen

**Affiliations:** a Centre for Medical Parasitology at Department of Immunology and Microbiology, Faculty of Health and Medical Sciences, University of Copenhagen, Copenhagen, Denmark; b Department of Immunology, Noguchi Memorial Institute for Medical Research, University of Ghana, Legon, Ghana; c Department of Epidemiology, Noguchi Memorial Institute for Medical Research, University of Ghana, Legon, Ghana; d Department of Infectious Diseases, Copenhagen University Hospital (Rigshospitalet), Copenhagen, Denmark; University of South Florida

**Keywords:** PfEMP1, *Plasmodium falciparum*, antibodies, immunity, malaria

## Abstract

Plasmodium falciparum erythrocyte membrane protein 1 (PfEMP1) is an important malaria virulence factor. The protein family can be divided into clinically relevant subfamilies. ICAM-1-binding group A PfEMP1 proteins also bind endothelial protein C receptor and have been associated with cerebral malaria in children. IgG to these PfEMP1 proteins is acquired later in life than that to group A PfEMP1 not binding ICAM-1. The kinetics of acquisition of IgG to group B and C PfEMP1 proteins binding ICAM-1 is unclear and was studied here. Gene sequences encoding group B and C PfEMP1 with DBLβ domains known to bind ICAM-1 were used to identify additional binders. Levels of IgG specific for DBLβ domains from group A, B, and C PfEMP1 binding or not binding ICAM-1 were measured in plasma from Ghanaian children with or without malaria. Seven new ICAM-1-binding DBLβ domains from group B and C PfEMP1 were identified. Healthy children had higher levels of IgG specific for ICAM-1-binding DBLβ domains from group A than from groups B and C. However, the opposite pattern was found in children with malaria, particularly among young patients. Acquisition of IgG specific for DBLβ domains binding ICAM-1 differs between PfEMP1 groups.

## Abstract (precis)

Plasmodium falciparum erythrocyte membrane protein 1 (PfEMP1) is an important malaria virulence factor. The protein family can be divided into clinically relevant subfamilies. ICAM-1-binding group A PfEMP1 proteins also bind endothelial protein C receptor and have been associated with cerebral malaria in children. IgG to these PfEMP1 proteins is acquired later in life than that to group A PfEMP1 not binding ICAM-1.

## INTRODUCTION

Plasmodium falciparum malaria is a major cause of morbidity and mortality among children in sub-Saharan Africa. Individuals living in areas with high-intensity transmission of P. falciparum acquire clinical immunity to the disease during childhood. The protection is mediated to a considerable extent by IgG specific for members of the P. falciparum erythrocyte membrane protein 1 (PfEMP1) family, expressed on the surface of infected erythrocytes (IEs) (reviewed in reference 1). PfEMP1 proteins are highly polymorphic and mediate IE adhesion to a variety of different receptors on endothelial cells (2, 3). The proteins are encoded by approximately 60 *var* genes, and transcriptional switching among these genes allows the parasite to change PfEMP1 expression and escape host antibodies (3, 4). This protects IEs harboring parasites from clearance by the spleen (5) and promotes survival and growth in the host (reviewed in reference 1). PfEMP1 proteins can be classified into three major groups (A, B, and C) based on sequence and chromosomal context of the *var* genes (6, 7). Parasite expression of group A PfEMP1 has repeatedly been associated with severe malaria (8, 9). Protective immunity to severe malaria is acquired before immunity to uncomplicated disease and asymptomatic infection (10, 11), and this is paralleled by acquisition of group A PfEMP1-specific IgG early in life (12, 13).

PfEMP1 proteins are characterized by their constituent Duffy-binding-like (DBL) and cysteine-rich interdomain region (CIDR) domains (2–4, 14). Particular subtypes of DBLβ and CIDRα domains have been associated with binding to endothelial receptors such as intercellular adhesion molecule 1 (ICAM-1), endothelial protein C receptor (EPCR), and CD36 (15–17). More recently, we identified particular group A PfEMP1 proteins that can bind both ICAM-1 and EPCR (18). The ICAM-1-binding DBLβ domains of such group A PfEMP1 proteins are characterized by a specific sequence motif, and IgG specific to them is acquired later in life than IgG specific for group A DBLβ domains that do not bind ICAM-1 (18, 19).

The acquisition pattern of ICAM-1-binding group B and C DBLβ-specific IgG is unknown. Therefore, the current study was designed to provide such data and to compare IgG reactivity to that of different subtypes of DBLβ domains in Ghanaian children with or without P. falciparum malaria. The aim was to provide increased understanding of how antibody-mediated immunity to PfEMP1 is acquired following natural exposure to P. falciparum.

## RESULTS

### Identification of ICAM-1-binding DBLβ domains.

**FIG 1:**
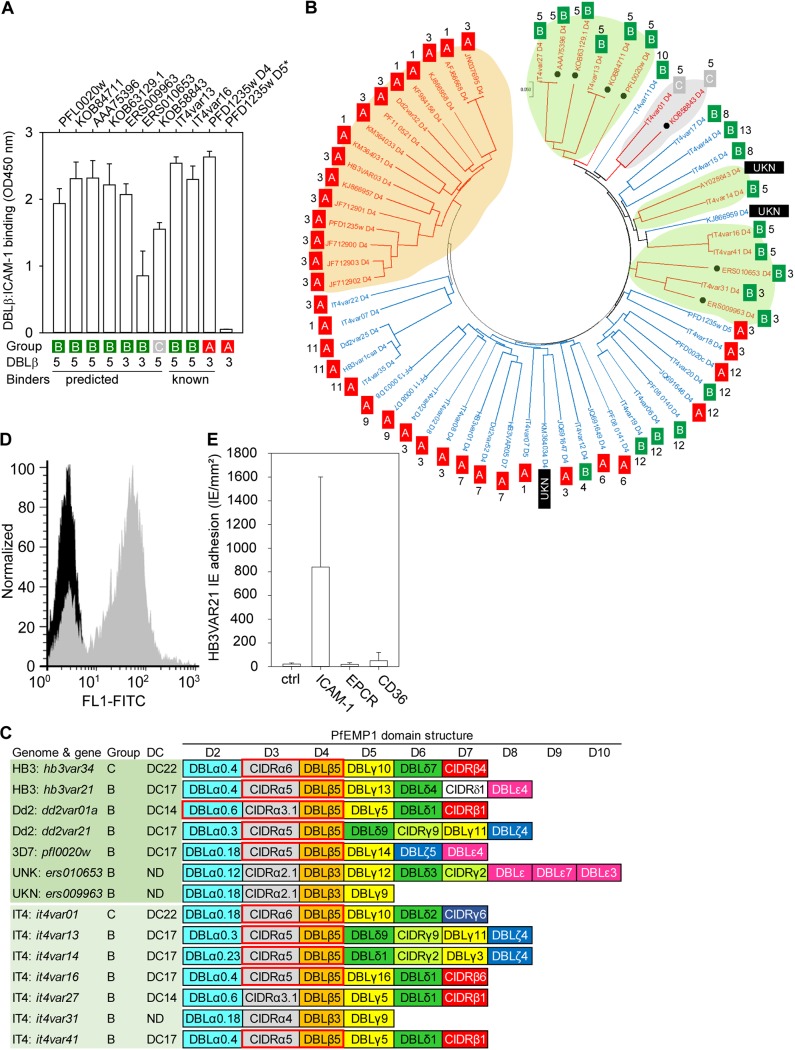
ICAM-1-binding DBLβ domains. (A) ICAM-1 binding (ELISA optical density at 450 nm [OD450 nm]; means ± SD from three independent experiments) of 11 DBLβ domains, seven of which were predicted to bind ICAM-1 (6 group B and 1 group C). PFD1235w_D4 (group A) and PFD1235w_D5 (*) were used as positive and negative controls, respectively. (B) Phylogeny of ICAM-1-binding (red gene names) and nonbinding (blue gene names) DBLβ, shown as a maximum likelihood tree of 62 DBLβ domains (18, 20, 54, 55). The new DBLβ domains tested in this study are indicated by black dots. UKN indicates unknown group identity. ICAM-1 binding DBLβ domains from groups A (orange shading), B (green shading), and C (gray shading) are highlighted. (C) Schematic domain structure of ICAM-1-binding group B and C PfEMP1 proteins, with domain cassettes (DC) indicated by red boxes. ICAM-1 binders identified in this study (dark green shading) and by Janes et al. (20) (light green shading) are also indicated. The classification and nomenclature of domain groups and subgroups follow those of Rask et al. (56). ND, not determined. (D) Surface expression of HB3VAR21 P. falciparum HB3 IEs, visualized by incubation with HB3VAR21-specific antiserum (gray) or without antiserum (black). (E) Ability of HB3VAR21^+^ IEs to bind to recombinant ICAM-1, EPCR, and CD36. Mean adhesion (three independent experiments) is shown, with SD indicated by error bars.

We have previously identified a novel family of group A ICAM-1-binding DBLβ domains associated with cerebral malaria (18). Here, we used BLASTP searches and amino acid sequences encoding ICAM-1-binding group B and group C DBLβ domains from P. falciparum IT4 (20) to search for additional DBLβ domains predicted to bind ICAM-1. Seven new sequences were identified by this approach. The encoded domains were a DBLβ3-type domain (GenBank accession no. KOB58843/HB3VAR34) and a DBLβ5-type domain (KOB63129/HB3VAR21) from HB3, two DBLβ5-type domains from Dd2 (AAA75396/Dd2VAR01A and KOB84711/Dd2VAR21), one DBLβ5-type domain from 3D7 (PFL0020w), and one DBLβ5-type domain from each of two field isolates (ERS009963 and ERS010653). Dd2VAR21/KOB84711 was identical to the previously published IT4VAR13, except for one residue (E instead of V) in DBLα and one residue (C instead of R) in the ATS region. All seven new domains bound ICAM-1 as predicted (Fig. 1A) and clustered together with other ICAM-1-binding DBLβ domains from groups B and C (Fig. 1B). The average sequence similarity of the new group B and C ICAM-1-binding DBLβ domains was 50%, which is comparable to that of previously identified ICAM-1-binding group A domains (58%) (18). Domains downstream of the ICAM-1-binding DBLβ domains belonged to groups and subgroups similar to those in the previously identified ICAM-1-binding group B and C PfEMP1 proteins (Fig. 1C). To validate these findings further, we immunized rats with one of the domains (HB3VAR21-DBLβ5_D4) and used the antiserum to select P. falciparum HB3 to express VAR21 on the surface of IEs (Fig. 1D). HB3VAR21^+^ IEs bound ICAM-1 at high levels (Fig. 1E), confirming the ability to predict the IE adhesion phenotype from *var* gene sequences.

### IgG specific for ICAM-1-binding group A DBLβ domains dominates in healthy children.

**FIG 2:**
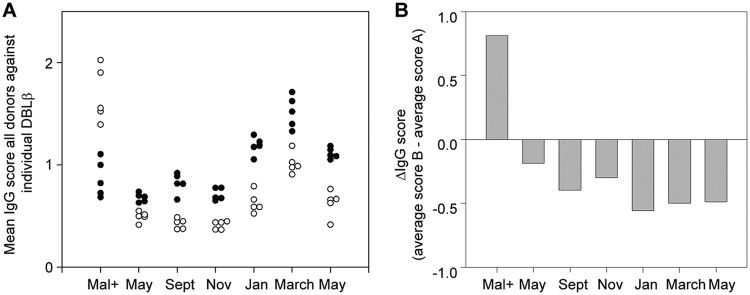
Difference in IgG responses to group A and B DBLβ domains. Blood samples were obtained from 124 Ghanaian children (labeled Mal+) 2 weeks after they were diagnosed with acute P. falciparum malaria and from 91 healthy children at six different time points over a 1-year period (May, September, November, January, March, and May). (A) Mean IgG scores in all donors against five DBLβ domains from group B (white circles; PFL0020w, KOB84711, AAA75396, ERS009963, and KOB63129) and five from group A (black circles; PFD1235w, JF712902, KJ866957, Dd2VAR32, and AFJ66668) are shown. (B) Difference (ΔIgG) in mean IgG scores against the same five group B and five group A recombinant DBLβ domains.

Group A PfEMP1-specific IgG is acquired earlier in life than antibodies targeting group B and C PfEMP1 antigens, and IgG to group A PfEMP1 therefore tends to dominate among healthy individuals living in areas with natural transmission of P. falciparum parasites (12, 21, 22). This was also the case here, when we compared IgG reactivity to ICAM-1-binding DBLβ domains in group A and group B PfEMP1 proteins, employing plasma from a cohort of healthy Ghanaian children. Because only small sample volumes were available for this testing, we selected five of the ICAM-1-binding group B DBLβ proteins identified above and five previously identified corresponding domains from group A (19). The IgG reactivity to each of the ICAM-1-binding DBLβ domains from group A was higher than the reactivity to any of the domains from group B. This was consistently the case with samples from the same donors but collected at six different time points over a 1-year period (Fig. 2; also see the data set in the supplemental material). This finding extends the earlier reports by demonstrating that the dominance of group A PfEMP1-specific IgG among healthy individuals remains when the comparison is restricted to ICAM-1-binding DBLβ domains only.

However, when we used plasma from children with acute P. falciparum malaria instead of plasma from healthy children, the pattern was the opposite, as levels of IgG specific for group B DBLβ domains were higher than those for group A domains (Fig. 2). This suggests that acute malaria episodes markedly perturb the steady-state (“healthy”) hierarchy of IgG reactivity to group A and group B PfEMP1 proteins, leading to a transient inversion of the group-specific IgG ratio. To examine this possibility further, we proceeded with a more detailed analysis of DBLβ-specific antibody responses, including kinetics and a larger panel of domains.

### The PfEMP1 group hierarchy of DBLβ-specific IgG is influenced by malaria episodes.

**FIG 3:**
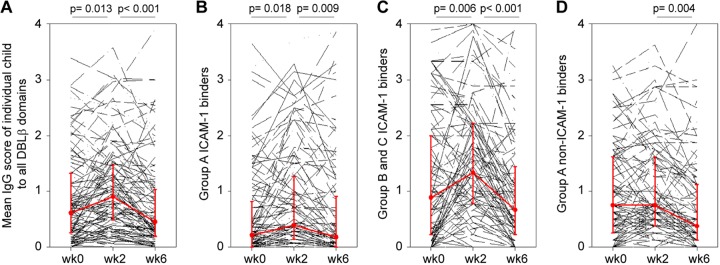
Individual mean IgG score of each child against DBLβ domains at admission (wk0) and two weeks (wk2) and six weeks (wk6) later. (A) Mean plasma IgG score of each individual Ghanaian child (*n* = 124) against a total of 31 group A, B, and C DBLβ domains. (B) Mean IgG scores in individual children against 14 ICAM-1-binding DBLβ domains from group A. (C) Nine ICAM-1-binding DBLβ domains from groups B and C. (D) Eight non-ICAM-1-binding DBLβ domains from group A. The wk0 data from 2014 in panels B and D were published in reference 19. Median levels (dots) and their percentiles (25% and 75%; error bars) are indicated in red. The statistical significance (Mann-Whitney rank sum test) of pairwise comparisons is shown along the top of each panel.

**FIG 4:**
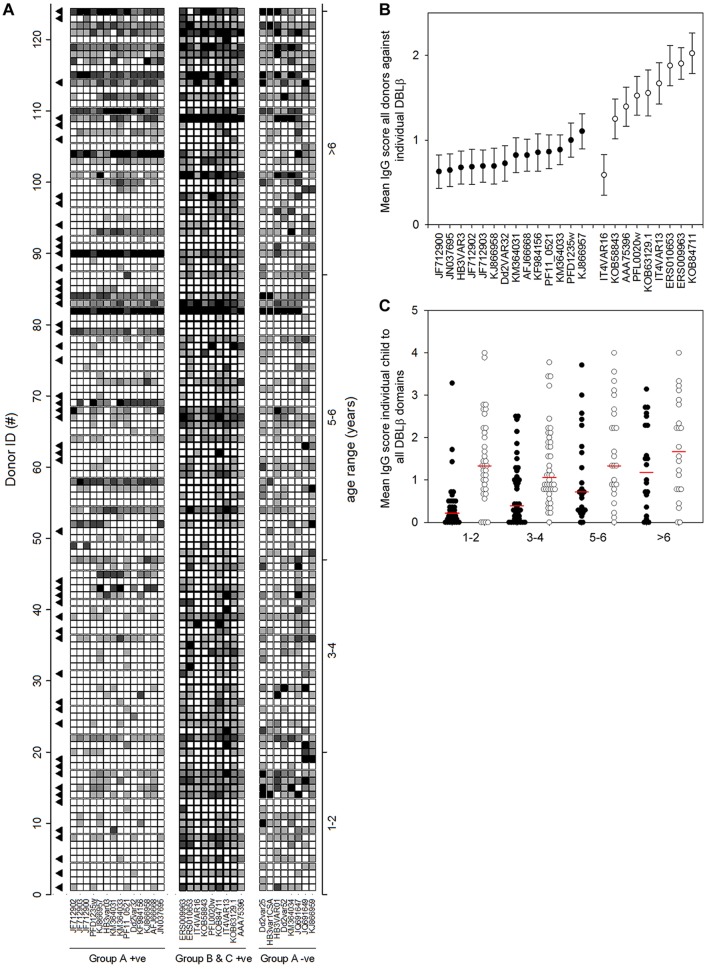
Plasma levels of IgG specific for DBLβ domains from group A, group B, and group C. Samples were obtained from 124 Ghanaian children diagnosed with severe (▲) or nonsevere P. falciparum malaria 2 weeks prior to taking the blood sample. (A) Levels of IgG antibodies specific for individual ICAM-1-binding DBLβ domains (columns) from group A (left) and groups B and C (center) or for non-ICAM-1-binding domains from group A (right). Shading indicates the IgG level score: black, 4; dark gray, 3; gray, 2; light gray, 1; white, 0. The domain subtypes are indicated in Table S1. Danish controls (*n* = 20) did not react with any of the domains (data not shown). (B) Mean week 2 scores of IgG specific for individual ICAM-1-binding DBLβ domains from group A (black) and groups B and C (white). Error bars indicate 95% confidence intervals. (C) Mean IgG scores against all DBLβ domains in individual children, grouped according to age. Medians are indicated by horizontal red lines.

Plasma levels of IgG to P. falciparum antigens, including PfEMP1, tend to increase in relation to malaria episodes among individuals with natural exposure to these parasites but decline again shortly after resolution of the infection (23, 24). The IgG responses to a large panel of DBLβ domains in Ghanaian children monitored over 6 weeks after acute P. falciparum malaria episodes showed a similar pattern. Responses were highly variable, and marked but mostly transient IgG responses to all the different types of DBLβ domains were observed in some but not all children (Fig. 3; also see the data set in the supplemental material). Nevertheless, the most prominent overall increase in DBLβ-specific IgG reactivity associated with malaria episodes was to group B and C antigens (Fig. 3C). This finding was underpinned by analysis of IgG responses to the individual DBLβ domains 2 weeks after admission, where responses generally peaked compared to the levels on admission and at week six (Fig. 3 and 4; also see the data set in the supplemental material). At that time, IgG reactivity to all but one of the ICAM-1-binding DBLβ antigens from group B and C PfEMP1 was higher than that to ICAM-1-binding group A domains (Fig. 4B). The difference between the two groups of PfEMP1 antigens was due to low IgG reactivity against group A DBLβ domains in children younger than 7 years (Fig. 4C). Furthermore, the IgG reactivity to group B and C domains did not differ between age groups (*P* = 0.5) (Fig. 4C), and significantly more of the children were seropositive for group B DBLβ-specific IgG (62 to 85%) than for corresponding domains from group A (30 to 60%) (*P* < 0.05) (Table S3). Overall, it appears that most of the clinical episodes involved parasite populations expressing a mixture of PfEMP1 variants, including proteins containing different types of DBLβ domains (Table S3), although parasites expressing group A ICAM-1-binding DBLβ domains seemed underrepresented among the younger children. Furthermore, our data suggest that prominent responses to group B and C DBLβ domains can cause a transient inversion of the ratio of IgG specific for ICAM-1-binding group A as well as group B and C DBLβ.

### IgG reactivity to ICAM-1-binding DBLβ domains in group A and groups B and C is similar in children with uncomplicated and those with severe, noncerebral malaria.

**FIG 5:**
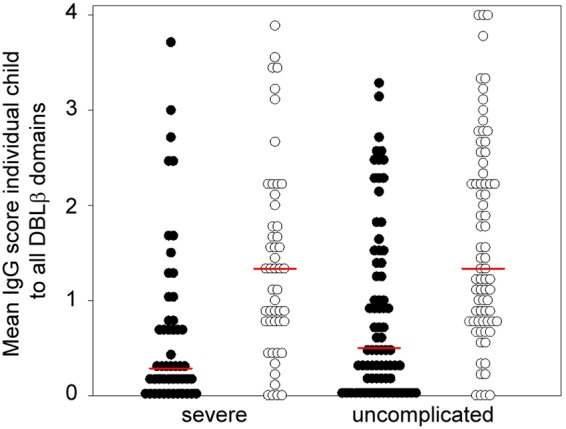
Mean IgG scores of 124 individual Ghanaian children against DBLβ domains from group A (black) and groups B and C (white) according to severity. Medians are indicated by horizontal red lines.

Previous studies have shown that IEs obtained from young children with severe malaria primarily express PfEMP1 encoded by group A *var* genes, while expression of PfEMP1 encoded by group B and group C *var* genes appears associated with uncomplicated disease in slightly older children (8, 25–27). We therefore proceeded to compare the DBLβ-specific IgG responses in children with severe malaria to those in children with uncomplicated malaria. IgG reactivity to ICAM-1-binding group B and C DBLβ domains was higher than that to corresponding group A domains when each group of patients was considered separately (Fig. 5; also see the data set in the supplemental material). However, no statistically significant differences were noted when IgG reactivity to ICAM-1-binding DBLβ domains from either group A or groups B and C was compared between children with severe or uncomplicated malaria (Fig. 5). While this may seem at variance with the well-documented relationship between expression of group A PfEMP1 and severe malaria, it should be noted that the subset of group A dual-receptor-binding PfEMP1 containing ICAM-1-binding DBLβ domains has been associated specifically with cerebral malaria and not severe malaria in general (18), and that only 2 of the 124 children studied fulfilled the criteria for such a diagnosis.

## DISCUSSION

P. falciparum causes the most severe form of malaria and is responsible for the vast majority of malaria-related deaths (28). This is not least due to the presence in this species of the PfEMP1 family of adhesive proteins, which are expressed on the surface of IEs in a mutually exclusive manner (only one variant expressed at a time) (4). Different members of the PfEMP1 family enable the adhesion of IEs to a range of vascular host receptors, which facilitate IE evasion of splenic clearance (5). It furthermore promotes tissue inflammation and organ dysfunction, while parasite switching among different PfEMP1 family members (variants) frustrates the development of protective PfEMP1-specific immunity (reviewed in reference 1).

Severe P. falciparum malaria has repeatedly been linked to IE adhesion to particular host receptors, mediated by groups and subgroups of structurally related group A PfEMP1 (reviewed in reference 1). Group A PfEMP1 proteins mediating adhesion to EPCR appear to be of particular relevance to the pathogenesis of severe malaria in patients with or without cerebral symptoms (8, 17, 25, 26, 29, 30). Cerebral malaria, one of the most severe complications of P. falciparum infection (reviewed in reference 31), is associated with expression of a subgroup of group A PfEMP1 variants with a dual-receptor adhesion phenotype (18, 29). These proteins carry an ICAM-1-binding DBLβ domain next to an EPCR-binding CIDRα1 domain. Although ICAM-1-binding DBLβ domains also occur in group B and C PfEMP1, these domains are structurally distinct, do not have a neighboring EPCR-binding but a CD36-binding CIDRα2-6 domain, and do not appear to play a role in cerebral malaria pathogenesis (18). Of the two domain types, CIDRα domains are more commonly recognized than DBL domains (12, 32), and some studies have shown acquisition of group A CIDRα1-specific IgG to precede immunity to group B and C CIDRα2-6 domains (33), while others found no such link (12, 34). Two recent studies found similar antibody reactivity against group A CIDRα1 in uncomplicated and severe malaria during acute disease (34, 35), while at convalescence older children with severe (likely noncerebral) malaria had higher antibody levels against such EPCR binding CIDRα1 than those with uncomplicated malaria (34). The PfEMP1 reactivity between convalescent groups did not differ in the study by Kessler et al. (35), although higher seroprevalence to the conserved group A-associated ICAM-1-binding DBLβ domain (18) was observed relative to that of CIDRα1.

In a longitudinal study assessing antibody acquisition against 32 non-ICAM-1-binding DBL domains (three α, eight β, five γ, nine δ, six ε, and one ζ), asymptomatic Tanzanian children were shown to acquire antibodies to group A prior to group B and C domains (13). In addition, it has recently been shown that the breadth of antibodies that inhibit adhesion of IEs to ICAM-1 increases with age in Malian children (36), although the study did not investigate whether these IEs expressed group A, B, or C PfEMP1 antigens. We find that among group A PfEMP1 proteins, acquisition of IgG to DBLβ domains that do not bind ICAM-1 appears to precede acquisition of IgG to those that do (19).

As the acquisition pattern of IgG specific for ICAM-1-binding DBLβ domains from groups B and C is not currently known, we first used a BLASTP search to extract new potential ICAM-1-binding DBLβ domains from these PfEMP1 groups. Seven such domains were identified and shown to bind ICAM-1 and to be structurally related to known ICAM-1-binding DBLβ domains from group B and C PfEMP1 (Fig. 1).

In healthy children, we found that IgG reactivity to ICAM-1-binding DBLβ domains from group A was higher than reactivity to corresponding domains from group B and C PfEMP1 (Fig. 2). Overall, these findings are in agreement with the previously reported dominance of responses to group A PfEMP1 over other PfEMP1 groups (12, 13, 37). However, when the assays were repeated with plasma obtained from children with acute malaria, we found higher overall IgG reactivity to ICAM-1-binding DBLβ domains from groups B and C rather than group A (Fig. 2). Furthermore, the most prominent change after acute malaria was a transient increase in IgG reactivity to ICAM-1-binding DBLβ domains from groups B and C (Fig. 3), and 2 weeks after the acute attack, reactivity to each of the group B and C ICAM-1-binding DBLβ domains was higher than that to the corresponding group A domains (Fig. 4). The most parsimonious explanation for the disease-related inversion of the ratio of IgG reactivity to ICAM-1-binding DBLβ domains from group A versus groups B and C is that the disease episodes in our study children were caused mainly by parasites expressing group B and C PfEMP1 or group A PfEMP1 without ICAM-1-binding DBLβ domains. This interpretation is consistent with the fact that only two of the study children were diagnosed with cerebral malaria (associated with parasites expressing group A DBLβ domains [18]). This in turn may explain why we did not observe differences in IgG reactivities between children of similar age with severe and uncomplicated malaria (Fig. 5). In agreement with this, a recent study found that children with uncomplicated and cerebral malaria had similar breadth and magnitude of responses to different P. falciparum antigens, including DBLβ domains (35). Finally, our data suggest that IgG specific for ICAM-1-binding DBLβ domains from group A and associated specifically with cerebral malaria (18) is acquired later in life than IgG specific for ICAM-1-binding DBLβ domains from group B and C PfEMP1 (Fig. 4C). We previously made a similar observation when IgG reactivity to ICAM-1-binding DBLβ domains in group A was compared to reactivity to DBLβ domains from the same group that do not bind ICAM-1 (19). Thus, the age where IgG specific for group A ICAM-1-binding DBLβ domains is acquired coincides with the age where cerebral malaria incidence peaks (38, 39). This is in striking contrast to the case for group A DBLβ domains that do not bind ICAM-1 (19) and for group B and C DBLβ domains that do (this study). However, whether a causal relationship exists remains to be investigated in a study area where the incidence of cerebral malaria is higher.

Opsonizing antibodies against PfEMP1 have been suggested to play a role in immunity to P. falciparum malaria (40), while other mechanisms, such as recruitment of complement (41), interaction with immune cells (42), and inhibition of vascular adhesion, might also play a role. Sequestration of P. falciparum IEs to the microvascular endothelium contributes to the pathogenesis of severe malaria in children, and broadly, cross-reactive antibodies inhibiting the interaction between ICAM-1 and DBLβ domains are detectable in immune plasma (18, 19, 29). We were unable to test these potential effector functions due to the limited plasma volumes available, but these are aspects that should be investigated in future studies.

In conclusion, our study demonstrates significant differences in the acquisition of IgG to ICAM-1-binding DBLβ domains from group A as well as group B and C PfEMP1. These differences are likely to be of significance in the development of PfEMP1-based vaccines to prevent severe P. falciparum malaria in general and cerebral malaria in particular (43).

## MATERIALS AND METHODS

### Study site and participants.

The study was conducted from 2014 to 2015 at Hohoe Municipal Hospital in the Volta Region of Ghana. Plasma samples were collected from children aged 1 to 12 years (*n* = 124) who reported with P. falciparum malaria (Table 1) (18, 19, 44). The inclusion criteria were a positive rapid diagnostic test for malaria, a positive blood smear of asexual P. falciparum parasites (>2,500/μl), and fever or a history of fever (>37.5°C) in the preceding 24 h. Patients were categorized as having severe malaria if they had unarousable coma (Blantyre coma score of ≤2) without other known causes, severe malarial anemia (hemoglobin of <5 g/dl), hyperparasitemia (≥250,000/μl), or respiratory distress (i.e., rapid, deep, and labored breathing) (45). Among the severe malaria patients, 15 children were diagnosed with respiratory distress, eight with severe anemia, and two with cerebral malaria. Children with uncomplicated malaria were cases without any of the severe disease symptoms; all children in this group were treated as outpatients. Blood samples were collected on the day of hospital admission and 2 and 6 weeks later. Patients receiving blood transfusion prior to follow-up were excluded from the study. Plasma collected from healthy children (*n* = 91) (Table 2) was also included in the study (46). A minority of those children had occasional, asymptomatic parasitemia.

**TABLE 1:** Clinical characteristics of Ghanaian study participants

Characteristic	Value by malaria type and age group^*a*^							
	Severe (*n* = 50)				Uncomplicated (*n* = 74)			
	1–2 yr (*n* = 16)	3–4 yr (*n* = 16)	5–6 yr (*n* = 13)	>6 yr (*n* = 5)	1–2 yr (*n* = 20)	3–4 yr (*n* = 25)	5–6 yr (*n* = 14)	>6 yr (*n* = 15)
Age (yr)	2.1 (1.7; 2.4)	4.2 (3.2; 4.5)	5.5 (5.3; 5.6)	7.8 (7.7; 8.3)	2.3 (1.8; 2.7)	4.1 (3.5; 4.3)	6.5 (5.4; 6.8)	9.0 (8.0; 10.7)
Blantyre coma score	5.0 (5.0; 5.0)	5.0 (3.3; 5.0)	5.0 (5.0; 5.0)	5.0 (3.0; 5.0)	5.0 (5.0; 5.0)	5.0 (5.0; 5.0)	5.0 (5.0; 5.0)	5.0 (5.0; 5.0)
Hemoglobin (g/dl)	8.6 (6.1; 10.0)	8.2 (7.4; 8.6)	9.1 (8.0; 10.6)	9.8 (7.9; 11.8)	10.0 9.4; 10.6)	9.8 (8.1; 11.2)	10.9 (9.5; 11.2)	11.2 (10.4; 11.8)
Parasitemia (no. of parasites/μl ×1,000)	87.8 (24.5; 152.8)	101.7 (8.8; 161.0)	52.2 (5.9; 148.7)	77.5 (49.2; 151.5)	42.5 (31.3; 85.3)	34.7 (12.3; 62.0)	23.5 (11.8; 82.8)	30.9 (13.0; 62.3)
Temperature (°C)	38.9 (38.1; 39.7)	38.1 (36.8; 39.2)	38.5 (37.5; 39.2)	38.4 (36.7; 38.6)	38.6 (37.5; 39.3)	38.4 (36.8; 39.5)	38.8 (38.2; 39.2)	38.6 (37.4; 39.6)

a Values are medians (25th; 75th percentile). Two study participants were diagnosed with cerebral malaria.

**TABLE 2:** Characteristics of healthy Ghanaian study participants

Characteristic	Value for year^*a*^:					
	1			2		
	May (*n* = 78)	Sept (*n* = 86)	Nov (*n* = 80)	Jan (*n* = 78)	March (*n* = 73)	May (*n* = 63)
Age (yr)	4 (3; 4.5)	4 (3; 4.5)	4 (3; 4.5)	4 (3; 4.5)	4 (2.5; 4.5)	4 (2.75; 4.5)
Hemoglobin (g/dl)	10.7 (9.6; 11.3)	10.1 (8.9; 10.9)	10.5 (9.5; 11.3)	10.8 (9.9; 11.7)	10.3 (9.3; 11.5)	11.0 (10.3; 11.9)
Parasitemia (no. of parasites/μl)	1,240 (560; 3,610)	2,400 (1,060; 7,930)	1,500 (510; 3,850)	2,080 (670; 4,970)	920 (360; 2,560)	1,000 (600; 2,320)
Temperature (°C)	37.1 (36.8; 37.2)	37.1 (36.8; 37.2)	37.0 (36.8; 37.1)	37.0 (36.7; 37.2)	37.1 (36.9; 37.3)	37.1 (36.9; 37.2)

a Values are medians (25th; 75th percentile).

The study was approved by the Ethical Review Committee of the Ghana Health Services (file GHS-ERC 08/05/2014).

### Identification of group B and group C DBLβ domains.

P. falciparum IT4 amino acid sequences encoding published ICAM-1-binding group B and group C DBLβ domains, *var01* (GenBank accession no. AAO67411), *var13* (ABM88750), *var14* (AAD03351), *var16* (AAS89259), *var27* (ABM88759), *var31* (AAF18980), and *var41* (ABM88768) (20), were used to extract by BLASTP search new potential ICAM-1-binding DBLβ domains from GenBank or from assemblies of Illumina whole-genome sequencing data as described previously (47).

### Production of recombinant proteins.

His-tagged DBLβ domains (see Table S1 in the supplemental material) were expressed in Escherichia coli Shuffle C3030 cells (New England Biolabs) from synthetic genes (https://eurofins.dk) or from DNA constructs generated by PCR from genomic DNA using specific primers (Table S2). The domains were purified by immobilized metal affinity chromatography (18, 19, 48). Recombinant ICAM-1-Fc (D1-D5 with a human Fc tag) was expressed in HEK293 cells and purified on a HiTrap protein G HP column (GE Healthcare) as described previously (48).

### ELISA.

Binding of ICAM-1-Fc to immobilized DBLβ domains and levels of antigen-specific IgG in human plasma (diluted 1:100) were assessed by enzyme-linked immunosorbent assay (ELISA) using MaxiSorp plates (Sigma-Aldrich).

The plates were coated with 22 group A and nine group B and C recombinant DBLβ proteins (2 μg/ml; overnight at 4°C). Sixteen domains were already known to bind ICAM-1 (18, 20), eight were already known not to bind ICAM-1 (19), and the remaining seven DBLβ proteins (groups B and C) were predicted to bind ICAM-1 based on the sequence analysis done in this study.

Binding of ICAM-1-Fc and human IgG antibodies to the immobilized DBLβ domains was detected using horseradish peroxidase-conjugated rabbit anti-human IgG (1:3,000; Dako) (18). Plates were developed using TMB PLUS2 (Kem-En-Tec) according to the manufacturer’s instructions. Optical density (OD) values were read at 450 nm using a VersaMax microplate reader (Molecular Devices). Plasma antibody reactivity was expressed in arbitrary ELISA units (EU) calculated by the equation (OD_sample_ − OD_background_)/(OD_positive control_ − OD_background_) × 100 (49) and translated into IgG level scores 0 (0 to 25 EU), 1 (26 to 50 EU), 2 (51 to 75 EU), 3 (76 to 100 EU), and 4 (>100 EU). A pool of P. falciparum-exposed Tanzanian individuals and 26 nonexposed Danish individuals were used as positive and negative controls, respectively. The mean (plus two standard deviation [SD]) value among the negative-control plasma samples was used as a cutoff value to define seropositivity.

### Generation of antiserum.

Rats were immunized with recombinant HB3VAR21-DBLβ_D4 as described previously (48). All experimental animal procedures were approved by The Danish Animal Procedures Committee (Dyreforsøgstilsynet) as described in permit no. 2013-15-2934-00920 and according to the guidelines described in Danish acts LBK 1306 (23/11/2007) and BEK 1273 (12/12/2005).

### Plasmodium falciparum parasite culture.

P. falciparum HB3 was maintained *in vitro* and selected with rat anti-HB3VAR21-DBLβ_D4 as described previously (48, 50). IE surface expression of PfEMP1 was regularly monitored by flow cytometry, and only cultures with more than 60% HB3VAR21^+^ IEs were used. The identity and clonality of the parasites used were routinely verified by genotyping as described previously (51). Mycoplasma infection was excluded regularly using the MycoAlert mycoplasma detection kit (Lonza) according to the manufacturer’s instructions.

### Flow cytometry.

P. falciparum IEs were DNA labeled with ethidium bromide and surface labeled with rat anti-HB3VAR21-DBLβ_D4 (1:20) and fluorescein isothiocyanate (FITC)-conjugated secondary rabbit anti-rat IgG (1:150; Vector Labs) as described previously (50). Fluorescence data from ethidium bromide-positive cells were collected on an FC500 MPL flow cytometer (Beckman Coulter) and analyzed using WinList, version 9.0 (Verity Software House).

### Parasite adhesion spot assay.

Falcon 1007 petri dishes were coated overnight (4°C) with recombinant CD36 (0.05 μg/spot), ICAM-1-Fc (0.5 μg/spot), or EPCR (0.1 μg/spot) in triplicates (48). Dishes were blocked (1 h) in phosphate-buffered saline (PBS) with 3% bovine serum albumin (BSA). Mature IEs were adjusted to 3% parasitemia and 1% hematocrit in RPMI 1640 supplemented with 2% normal human serum, added to the dishes, and incubated (37°C, 30 min) as described previously (52). After removal of nonadherent IEs by sequential washing using prewarmed RPMI wash buffer, the remaining cells were fixed in 1.5% glutaraldehyde (15 min) and stained with Giemsa. After rinsing with water, the dishes were air dried overnight before the number of adherent IEs per square millimeter was quantified using ImageJ software (http://rsb.info.nih.gov/ij/). A minimum of three independent experiments in triplicate were done. All assays were blinded.

### PfEMP1 sequence similarity and phylogenetic trees.

The Praline multiple-sequence alignment tool (http://www.ibi.vu.nl/programs/pralinewww/) was used to calculate the average amino acid similarity of DBLβ domains. Multiple alignments of DBLβ domains were made using Muscle (https://www.ebi.ac.uk/Tools/msa/muscle/) and analyzed using Mega 5.0 software (53) to create cladograms.

### Statistics.

We used Kruskal-Wallis one-way analysis of variance on ranks and Mann-Whitney test to assess intergroup differences. Data analysis was done using SigmaPlot 13.0 (Systat Software Inc., United Kingdom).

## Supplementary Material

Supplemental file 1

Supplemental file 2
