# Supplementary material for: Acquisition of IgG to ICAM-1-Binding DBLβ Domains in the Plasmodium falciparum Erythrocyte Membrane Protein 1 Antigen Family Varies between Groups A, B, and C
Source: Infect Immun. 2019 Sep 19;87(10):e00224-19. doi: 10.1128/IAI.00224-19 (PMC6759304; doi:10.1128/IAI.00224-19)
Supplement: Supplemental file 1 [file IAI.00224-19-s0001.pdf]

Supplemental Material

Table S1. Recombinant proteins used in the study.

Genome	PfEMP1	Domain subtype	Binds ICAM-1	*var* group
BM048	JF712902	DBLβ3_D4	Yes[a]	A
BM066	JF712903	DBLβ3_D4	Yes[a]	A
BM021	JF712900	DBLβ3_D4	Yes[a]	A
3D7	PFD1235w[b]	DBLβ3_D4	Yes[a]	A
MN35	KJ866957	DBLβ3_D4	Yes[a]	A
HB3	HB3var03	DBLβ3_D4	Yes[a]	A
MN56	KM364031	DBLβ3_D4	Yes[a]	A
	KM364033	DBLβ3	Yes[a]	A
3D7	PF11_0521	DBLβ3_D4	Yes[a]	A
Dd2	Dd2var32[c]	DBLβ3_D4	Yes[a]	A
MN062	KF984156	DBLβ1	Yes[a]	A
A4395	KJ866958	DBLβ3	Yes[a]	A
1914	AFJ66668	DBLβ3_D4	Yes[a]	A
BM057	JN037695	DBLβ3_D4	Yes[a]	A
	ERS009963	DBLβ	Yes	B
	ERS010653	DBLβ	Yes	B
IT4	IT4var16[d]	DBLβ5	Yes[e]	B
HB3	HB3var34[f]	DBLβ5	Yes	C
3D7	PFL0020w[g]	DBLβ5	Yes	B
Dd2	Dd2var21[h]	DBLβ5	Yes	B
IT4	IT4var13[i]	DBLβ5	Yes[e]	B
HB3	HB3var21[j]	DBLβ5	Yes	B
Dd2	Dd2var01a[k]	DBLβ5	Yes	B
Dd2	Dd2var25	DBLβ11	No[a]	A
HB3	HB3var1CSA	DBLβ11	No[a]	A
HB3	HB3var01	DBLβ7	No[a]	A
Dd2	Dd2var52	DBLβ7	No[a]	A
MN35	KM364034	DBLβ6	No[a]	A
1983	JQ691647	DBLβ3	No[a]	A
1983	JQ691649	DBLβ6	No[a]	A
A4393	KJ866959	DBLβ3	No[a]	A

[a]Data from [18] Also known as PF3D7_0425800
[b] Also known as PF3D7_0425800
[c]Also known as KOB85388
[d]Also known as AAS89259
[e]Data from [23]

[f]Also known as KOB58843
[g]Also known as XP_001350413
[h]Also known as KOB84711
[i]Also known as ABM88750
[j]Also known as KOB63129.1
[k]Also known as AAA75396

Table S2. Primers for cloning of new ICAM-1 binding Group B and C DBLβ domains

Genome	Gene ID	Domain	Primers (forward/reverse)	Restriction enzymes
3D7	*PFL0020w*[a]	DBLβ5	GCGGATCCAATCCGTGTAGCGCCCAACCTG<br>CTAGGATCCTTAACACTCACATGCGTCCTTATAC	*BamH*I<br>*BamH*I
HB3	*HB3var34*[b]	DBLβ5	GAAGATCTAATGCTTGTAGTGGAGACC<br>CTAGCTAGCTTATCCACACGCATCTTCATATC	*Bgl*II<br>*Nhe*I
	HB3var21[c]	DBLβ5	GCGGATCCAATCCTTGTGCAGACAAAAATG<br>AGCTCGAGTTAACACCCACATGCCTTTTCATAGTC	*BamH*I<br>*Xho*I
Dd2	*Dd2var21*[d]	DBLβ5	GCGGATCCAATCCCTGTGTAGTTGGAGGC<br>CTAGCTAGCTTATTAACATTTACATGCATTAGCATA	*BamH*I<br>*Nhe*I
	Dd2var01a[e]	DBLβ5	GCGGATCCAATCCATGTAGTGGCGAAAGTGGTA<br>AGCTCGAGTTAGCAATCACACGCTGTAGCATA	*BamH*I<br>*Xho*I
Synthetic gene	*ERS009963*	DBLβx	GCGGATCCAACCCCTGTGGCAAAACCGAT<br>AGCTCGAGTTAGCTAGCGCACGCACATGCTTTC	*BamH*I<br>*Xho*I
	ERS010653	DBLβx	GCGGATCCAACCCGTGTGCAAAACCCGA<br>AGCTCGAGTTAGCTAGCGCATTCGCACGCTTTCT	*BamH*I<br>*Xho*I

[a]Also known as XP_001350413
[b]Also known as KOB58843
[c]lso known as KOB63129.1
[d]Also known as KOB84711
[e]Also known as AAA75396

Table S3. Individuals with positive seroreactivity against DBLβ domains

DBLβ domain	Group	Seroprevalence (%)[a] All (n=124)	1-2[b] (n=20)	3-4[b] (n=27)	5-6[b] (n=40)	>6[b] (n=37)
JF712902	A	35	45	48	30	16
JF712903	A	39	75	70	38	30
JF712900	A	30	65	37	33	14
PFD1235w[c]	A	55	75	63	45	30
KJ866957	A	60	60	63	35	38
HB3var03	A	32	65	63	35	19
KM364031	A	41	60	41	35	16
KM364033	A	48	70	63	48	49
PF11_0521	A	46	65	41	38	24
Dd2var32[d]	A	41	65	52	35	27
KF984156	A	43	65	70	25	46
KJ866958	A	34	60	37	25	14
AFJ66668	A	49	65	37	33	16
JN037695	A	33	55	59	38	30
ERS009963	B	73	50	37	23	35
ERS010653	B	85	75	78	55	46
IT4var16[e]	B	77	70	70	53	65
KOB58843	C	62	60	48	53	51
PFL0020w[f]	B	71	70	63	35	59
KOB84711	B	85	55	52	48	57
IT4var13[g]	B	34	65	52	33	30
KOB63129	B	77	70	74	60	68
AAA75396	B	68	60	70	55	54
Dd2var25	A	52	80	89	83	86
HB3var1CSA	A	41	65	78	60	70
HB3var01	A	59	80	78	73	81
Dd2var52	A	63	85	67	65	73
KM364034	A	54	70	63	58	62
JQ691647	A	67	80	81	68	84
JQ691649	A	60	75	74	65	78
KJ866959	A	52	85	85	83	89

[a]Percentage of individuals with a positive plasma sample above cut-off (DK samples + 2SD)
[b]Age bracket (years-of-age)
[c]Also known as PF3D7_0425800
[d]Also known as KOB85388
[e]Also known as AAS89259
[f]Also known as XP_001350413
[g]Also known as ABM88750
